# Evolutionary constraints shape the diversity of microinsects’ wing morphology

**DOI:** 10.1098/rspb.2025.1754

**Published:** 2025-11-05

**Authors:** Jakub Białkowski, Jostein Gohli, Robert Rossa, Anna Ziemiakowicz, Jakub Goczał

**Affiliations:** ^1^ Department of Forest Ecosystem Protection, University of Agriculture in Krakow, Krakow 31-425, Poland; ^2^ Norwegian Institute for Nature Research, Oslo NO-0855, Norway

**Keywords:** miniaturization, wing, feather-like, bristle, flight, insect, evolution, phylogenetic constraints, adaptation, bristled wing

## Abstract

Miniaturization profoundly alters animal morphology, particularly locomotory structures like insect wings. Larger insects possess membranous wings optimized for flight dominated by inertial forces, while microinsects have highly derived bristled wings with a reduced membrane, adapted to viscous interactions. Distantly related microinsects share striking similarities in some aspects of wing architecture, such as elongated bristles or narrowed wing blades, while features such as venation or proportion of bristled wing area vary widely. The basis of these differences remains unknown. Although insect wing morphology is largely shaped by evolutionary history, the role of evolutionary constraints in macro-to-micro wing transition has not been examined. For the first time, we combined morphological analyses with evolutionary modelling to explore how selection for wing optimization during miniaturization is constrained by evolutionary inertia in key wing features. Analysing 39 bark beetle species, ranging greatly in size, we found that some modifications, like bristle elongation or wing narrowing, exhibit very low evolutionary constraints, enabling rapid adaptation to miniaturization. In contrast, traits like venation development or bristled area proportion were highly constrained, requiring longer evolutionary timescales to adapt. Our findings provide novel insights into the origins of wing-architecture diversity in microinsects, emphasizing the role of evolutionary constraints in modulating the transition from macro- to micro wings.

## 1. Introduction

Over the past 350 Ma, winged insects have undergone diverse evolutionary changes, shaped by shifting selective pressures and constrained by evolutionary limits [1–3]. This evolutionary journey has ultimately resulted in a remarkable diversity of wing forms, ranging from the large, robust wings of the extinct †Meganisoptera (Odonatoptera), with wingspans reaching up to 71 cm [2], to the tiny, bristled micro wings of featherwing beetles (Ptiliidae), measuring just 0.1 cm in wingspan [4]. Among the most significant challenges in insect wing evolution was thus the fundamental reorganization of wing architecture necessitated by the gradual process of body miniaturization. This process—where extremely tiny species emerge within lineages descended from larger ancestors [5]—has independently occurred across numerous insect orders, including wasps, flies and beetles [6]. Among these, beetles (Coleoptera) stand out for their extraordinary range of body sizes, from the colossal Titan beetle (>17 cm) to the minute featherwing beetles (<0.04 cm for some species) [7,8]. Remarkably, even at these extremes of size, beetles retain fully functional wings capable of active flight, underscoring the evolutionary ingenuity required to optimize flight efficiency across vast size scales.

Insect flight is largely influenced by body size, which affects the physical conditions under which wings operate. Larger insects fly at a high Reynolds number regime, where inertial forces dominate, while microinsects operate at a low Reynolds number, relying primarily on viscous interactions [9]. This shift in aerodynamic regime poses major biomechanical challenges for the smallest flying insects. Notably, the aerodynamic force produced by flapping wings decreases faster than body weight as size diminishes, making it increasingly difficult for microinsects to generate sufficient lift. One common evolutionary response is the development of disproportionately large wings. However, such large wings may be physiologically costly for microinsects to develop, power and maintain. As a result, various compensatory strategies have evolved. These often involve modifications of wing shape that enable the generation of substantially greater aerodynamic force compared with unmodified wings of the same size [4,9–11]. Another key challenge for microfliers is overcoming the high viscous drag induced by the air surrounding their wings. One remarkable solution is to replace a solid membrane with a bristled wing surface (ptiloptery), which significantly helps reduce the inertial costs of flight [4].

The flight wings of larger insects typically consist of a solid membrane reinforced by a robust venation system, providing both structural integrity and aerodynamic efficiency. In contrast, the wings of the smallest insects, particularly those under 2 mm of body length, often adopt a racket-shaped form (i.e. resembling the outline of a tennis racket) and a feather-like structure composed of elongated bristles attached to a highly reduced membrane—a condition known as ptiloptery [10,12–14]. Notably, ptiloptery has independently evolved to varying degrees multiple times across distantly related insect lineages [6]. However, the pathways leading to this adaptation, and the role of evolutionary constraints in this modification, remain poorly understood.

Existing studies are mostly descriptive, focusing predominantly on clades in which all members are small-sized, which hinders a deeper understanding of the initial phases of wing miniaturization and the key morphological preconditions facilitating these transitions. Furthermore, although members of distinct microinsect groups exhibit some noticeable similarities in wing morphology, such as elongated marginal bristles, reduced membrane and narrowed proximal wing part (‘racket-shaped wing blades’), they also significantly differ in other wing elements (e.g. the level of wing vein development, bristle number and percentage of bristled area) [10,11,14–16]. As such, the nature of this diversity requires further investigation [11,15]. Wing morphology is known to be influenced by phylogenetic history [17,18], as shown by the high level of conservation in several wing features [17]. However, the role of evolutionary constraints in wing adaptation to decreasing body size has, surprisingly, not been comprehensively analysed. To date, no phylogenetic comparative analysis has rigorously evaluated the effect of ancestral stages on the process of miniaturization of insect wings. Such an analysis is crucial for understanding the interplay between functional demands and evolutionary inertia in shaping the diverse wing architectures observed in microinsects.

For the first time, we integrate advanced evolutionary modelling with detailed morphological analyses to investigate the critical modifications of insect wing morphology associated with progressive body miniaturization. Our study focuses on 39 species of bark beetles and pinhole borers (Curculionidae: Scolytinae, Platypodinae), spanning a wide range of body sizes from 8.7 to 1.2 mm. We chose bark beetles as a model group because they represent a relatively young, diverse and monophyletic lineage with a relatively well-resolved phylogenetic background [19]. Crucially, this group spans a range of body sizes where significant modifications in wing morphology are expected to occur [9,11]. Furthermore, unlike many other insect groups (e.g. flies or wasps), the flight apparatus of bark beetles is probably not involved in other behavioural contexts, which in other taxa may exert significant selective pressure and affect wing evolution.

Our extensive dataset allows us to capture a crucial sequence of wing modifications, from the initial emergence of marginal bristles through successive stages of wing shape modification and bristle elongation, culminating in the development of highly derived, ‘racket-shaped’ micro wings characterized by shortened veins, reduced membrane and long marginal bristles.

In our analysis, we evaluated the effect of phylogenetic constraints on the adaptation to body-size reduction, focusing on seven key wing parameters that previous studies [3,9–12,14,18] identified as changing markedly during body-size decrease: (i) bristled area, (ii) bristle length, (iii) wing aspect ratio, (iv) bristled region percentage, (v) vein divergence, (vi) veins length and (vii) bristle number.

## 2. Materials and methods

### (a) Studied taxa

We analysed 39 species of bark beetles and pinhole borers (Curculionidae: Scolytinae, Platypodinae), representing a broad range of body sizes, from 8.7 to 1.2 mm in length. Whenever possible, both wings from at least two males and two females per species were examined. However, the count of marginal bristles along the wing edge was based on only two specimens per species. Specimens were randomly selected from the insect collection at the Department of Forest Ecosystem Protection, Faculty of Forestry, University of Agriculture in Krakow.

### (b) Sample preparation and morphological measurements

**Figure 1.**
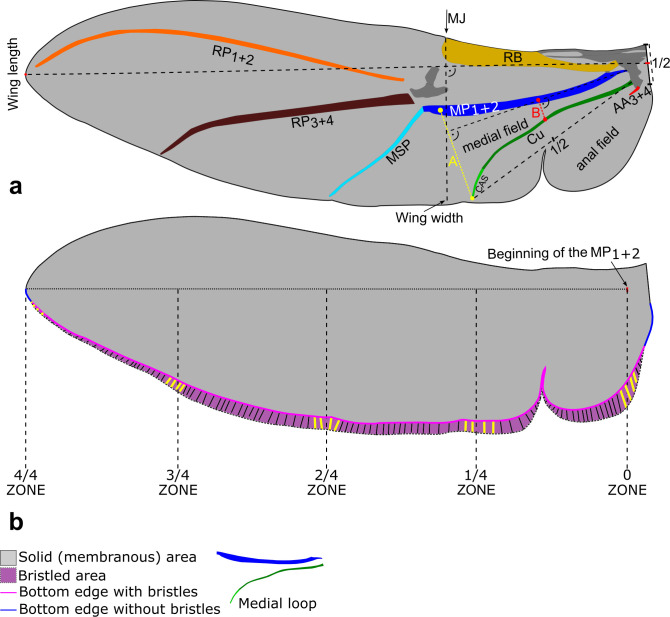
Morphological measurements of bark beetle wings. (a) Length of wing veins (AA_3+4_, anal anterior; Cu, cubital (including CAS, cubitoanal strut); MP_1+2_, media posterior; RB, radial bar; MSP, medial spur; RP_3+4_, radius posterior (branches 3 and 4); RP_1+2_, radius posterior (branches 1 and 2); MJ, marginal joint; A, distance between the media posterior vein and the cubital vein (+cubitoanal strut) measured at the end of the Cu+CAS; B, distance between the media posterior vein and the cubital vein (+cubitoanal strut) measured in the half of the Cu+CAS; wing length, from the centre of the wing base to the wing tip; wing width, from the MJ to the bottom edge measured perpendicular to the horizontal axis of the wing. (b) Solid (membranous) wing area, grey zone; bristled area, purple area; bristle length, length of the four bristles closest to the wing section lines (indicated in yellow); length of the bristled part of the bottom wing edge, pink line; length of the bristless part of the bottom wing edge, blue line.

Specimens were identified to species and sex [19–21] before analysis. Each individual was photographed using the Keyence VHX-7000 4K high-accuracy digital microscope (Keyence, Japan). Wings were carefully removed from each specimen using microsurgical tools, washed in pure ethanol, placed on a microscope slide and gently straightened in a drop of ethanol using a soft microbrush before preparations were made for imaging. Wing preparations were subsequently photographed using the VHX-7000 microscope. Body length (from the top of the pronotum to the apex of the elytra), wing length and wing width (see figure 1a for details) were measured based on obtained body and wing images. Subsequently, seven parameters of wing architecture previously considered to be strongly affected by miniaturization were analysed:

(1) Bristled area: the percentage of the bristled area relative to the total wing area (see figure 1b).
(2) Bristle length: the mean length of marginal bristles at the central region of the wing (zones 2/4 and 3/4; see figure 1b). Although the bristle length was measured in four wing zones (see figure 1b), zones 2/4 and 3/4 were chosen for analysis as they were the most representative for the analysed groups. Bristles occur in this region even in larger species, allowing for the observation of gradual changes with decreasing size.
(3) Bristle number: the total number of bristles divided by the wing length (to obtain a size-independent relative variable).
(4) Bristled region percentage: the percentage of the bottom wing edge (trailing edge) covered with bristles relative to the total length of the bottom wing edge (see figure 1b).
(5) Wing aspect ratio: the ratio of wing length to wing width (figure 1a; see [22] for methodological details).
(6) Vein divergence: measured as the distance between the media posterior and the cubital veins (including the cubitoanal strut), calculated by subtracting distance B from distance A (see figure 1a). Both distances A and B were divided by the wing length to obtain a size-independent relative variable.
(7) Vein length: measured as the relative sum of vein length (sum of vein length divided by wing length); see figure 1a.

Measurements were conducted using Digimizer v. 6.3.0 software (MedCalc Software Ltd, Belgium). For each species, values from multiple specimens were averaged to obtain a single species-level mean per trait used in comparative analyses.

### (c) Phylogenetic reconstruction

We started with a genera-level phylogenetic topology from the literature [23], which was based on five genes (*COI*, *EF-1a*, *28S*, *CAD*, *ArgK*). All genera not represented in our dataset were pruned from this phylogeny before missing genera were coded in, based on other published phylogenies [24]. Genera with only one representative species in our data were assigned the appropriate species names. Genera with two species representatives were added as sister species, while the relationship between species from genera with more than two species representatives was obtained from the literature [25–29] before coding these manually into our phylogeny.

The node heights of the phylogeny were inferred by analysing *COI* sequences downloaded from BOLD in BEAST v. 2.7.6 [30]. *Cryphalus intermedius* sequences, of which none was published in BOLD, were obtained from the authors of the publication focused on the genus *Cryphalus* taxonomy [27]. Only the node heights were sampled during the BEAST analysis—the topology, which was obtained from literature, remained fixed. Based on the analysis of our alignment in ModelTest-NG [31], we used a general time reversible site model, with the gamma category count set to 4, and without estimation of invariant sites. The analysis was performed with a Yule tree prior and a relaxed molecular clock.

### (d) Phylogenetic comparative analyses

For our phylogenetic comparative analysis of wing traits, we applied the SLOUCH (Stochastic Linear Ornstein–Uhlenbeck Models for Comparative Hypotheses) method [32], implemented in R (v.4.4.1) [33]. SLOUCH generates two models:

*The null model (intercept-only model)*: This model does not include any predictor variables and assesses the phylogenetic effect on a trait. It estimates how long it takes for a trait to lose half of its correlations with the ancestral trait value. If the *intercept-only half-life* (measured in tree length units) is zero, the trait is not influenced by ancestral conditions. An intercept-only half-life greater than zero indicates that ancestral influences persist, with the strength of this effect proportional to the intercept-only half-life value. For example, a half-life of 0.5 implies that half of the evolutionary history of the group is needed for the trait to lose half of the correlation with the ancestral trait value. If the intercept-only half-life exceeds 30 times the phylogeny length, the phylogenetic effect is so strong that the model approximates Brownian motion (diffusion with negligible pull towards an optimum).

*The regression model*: This model includes a predictor variable (selection driver), assuming the trait (response variable) evolves towards an *adaptive optimum* under selection. The adaptive optimum is the state where the trait achieves perfect adaptation to the predictor (selection driver; in our case this is the body size regime), ancestral constraints have disappeared and selection pressure from this particular driver drops to zero. In our specific case, the *adaptive optimum* for a given wing parameter (e.g. bristle length) is the state in which that parameter is perfectly adapted (i.e. bristles have the optimal length) to the prevailing body-size regime (with body size acting as the selection driver), so that no further selection for optimization of bristle length to body size occurs. Any delay in reaching this adaptive optimum is measured by the phylogenetic half-life, indicating how long it takes for the trait to evolve halfway towards adaptation. A phylogenetic half-life of zero means the trait adjusts instantly to the given predictor. A phylogenetic half-life of 1 implies that the full evolutionary history of the group is required for halfway adaptation to adaptive optimum. The larger the half-life estimate, the stronger the phylogenetic inertia. The regression model produces an evolutionary and optimal regression slope. The evolutionary regression slope shows the observed relationship between the predictor and the response variables, while the optimal slope shows the expected relationship in a scenario with no constraints on adaptation, i.e. the optimal adaptive values in the response variable as a function of the predictor.

Since the half-life parameter in SLOUCH is expressed as a proportion of phylogeny age, we set the root depth of our phylogeny to 1. This means that all estimates of half-life can be read as a percentage of the group’s evolutionary history. We also supply half-life parameters in Myr; the root age of our phylogeny was set to 105 Ma, which is based on the best available estimate for the split between Scolytinae and Platypodinae [34]. The root age used corresponds closely with a previously published phylogenetic study, which suggests approximately 100 Ma [35].

The null model and the regression model, for each of the seven wing traits (with body size (body length) as the singular predictor variable, i.e. the selection driver), were compared using δAICc (differences in the Akaike information criterion corrected for small sample size). When a regression model had an AICc score of >2 smaller than the corresponding null model, the regression model was considered statistically significant [36].

## 3. Results

### (a) Marginal bristles

**Figure 2.**
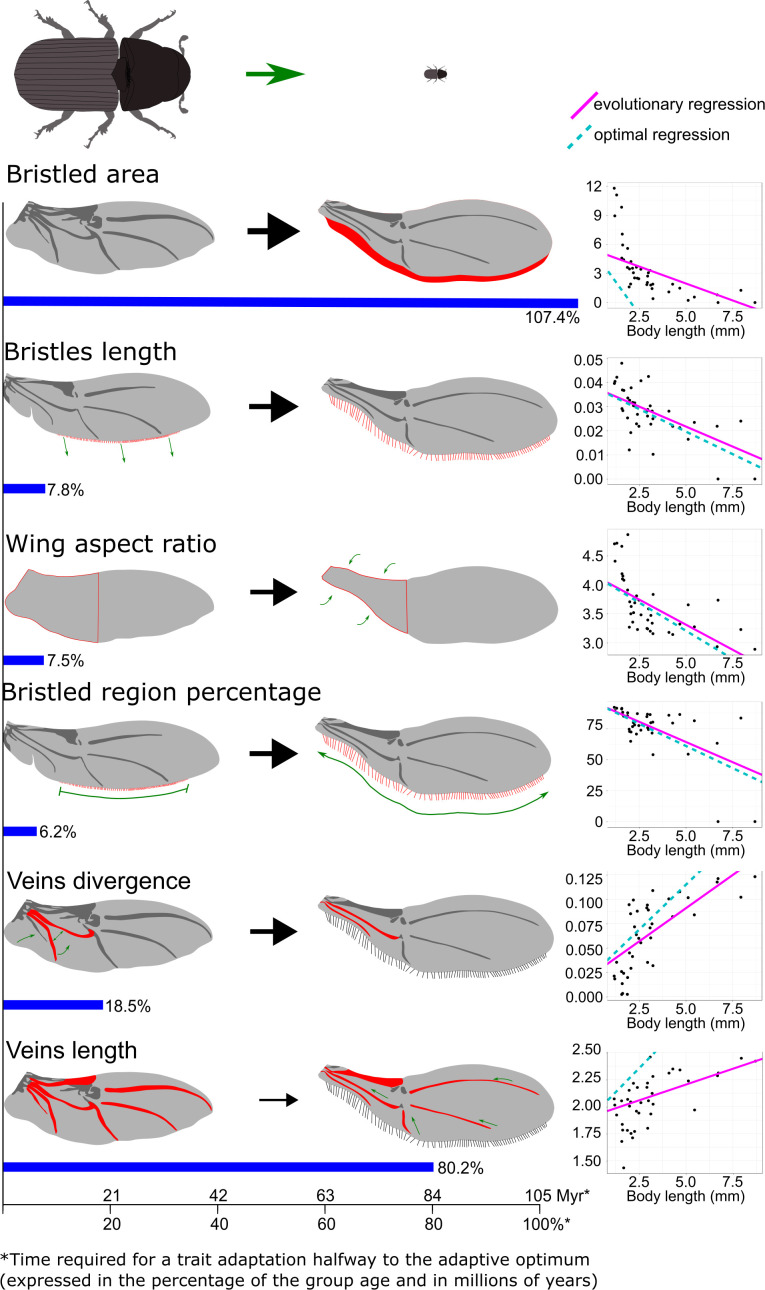
Diagram illustrating key changes in wing architecture associated with body miniaturization. Black arrows represent the strength of miniaturization effects, proportional to the coefficient of determination. Blue bands indicate phylogenetic inertia in adaptations to body-size reduction. Scatter plots show the difference between optimal regression slopes (unbiased by ancestral stages) and evolutionary (actual) regression slopes. Beetle drawings depict the actual scale of the size differences between the largest and the smallest bark beetle species included in the analysis.

The proportion of the bristled wing area to the solid (membranous) wing area increased significantly as body size decreased (table 1, figure 2), ranging from 0% in *Dactylipalpus* sp. (mean body length = 8.7 mm) to 12% in *Crypturgus pusillus* (mean body length = 1.2 mm). The greatest increase in bristled region was observed in the proximal part of the wing, primarily in the region (anal field) where the membrane surface experienced the most significant reduction (figure 2). The bristled area was strongly influenced by body miniaturization (figure 2) but exhibited a slow adaptation response to changes in body size owing to evolutionary constraints (table 1, figure 2). This is highlighted by the steeper slope of optimal regression compared with evolutionary regression (figure 2). More than 100% of the entire phylogenetic history of the group would be required to optimize this parameter halfway to its adaptive optimum (table 1). Body size explained a substantial portion of the variance in bristled area (*R*² = 0.37; table 1).

**Table 1.** Results from SLOUCH models for each of the seven wing parameters. BL, body length; SV, stationary variance. *AICc values from SLOUCH regression models compared with null models. δAICc is the difference in AIC value between the null and the regression model. δAICc values <− 2 are considered significant and are shown in bold in the table. The half-life is expressed as a proportion of the clade’s age (set to 1). A phylogenetic half-life of 1 indicates that the trait requires the clade’s entire evolutionary history to move halfway towards its adaptive optimum.

response	pred.	*n*	intercept-only half-life	phylo. half-life	SV	*R* ^2^	AICc	δAICc*
bristled area	BL	39	0.678	1.074	5.78	0.371	167.0	**−14.74**
vein length	BL	39	0.662	0.802	0.04	0.348	−24.3	**−15.05**
bristle length	BL	39	0.178	0.078	0.0001	0.386	−262.0	**−14.77**
vein divergence	BL	39	0.198	0.185	0.0005	0.507	−170.0	**−25.84**
bristled region percentage	BL	39	0.073	0.062	204.0	0.390	329.0	**−16.71**
bristle number	BL	39	2.415	2.416	1977.6	0.003	358.0	2.37
wing aspect ratio	BL	39	0.187	0.075	0.15	0.381	47.0	**−16.11**

As body size decreased, the percentage of the bottom wing edge covered with bristles gradually increased. In the smallest species (*C. pusillus*), marginal bristles were present along nearly the entire length of the bottom wing edge (92.1% of the length covered). In contrast, the largest species (*Dactylipalpu*s sp*.*) had no bristles on the bottom wing edge. The SLOUCH model revealed that miniaturization had a considerable effect on this trait (see the regression slope, figure 2), and the phylogenetic constraints on this trait were low, entailing fast adaptation to body-size reduction (table 1, figure 2).

**Figure 3.**
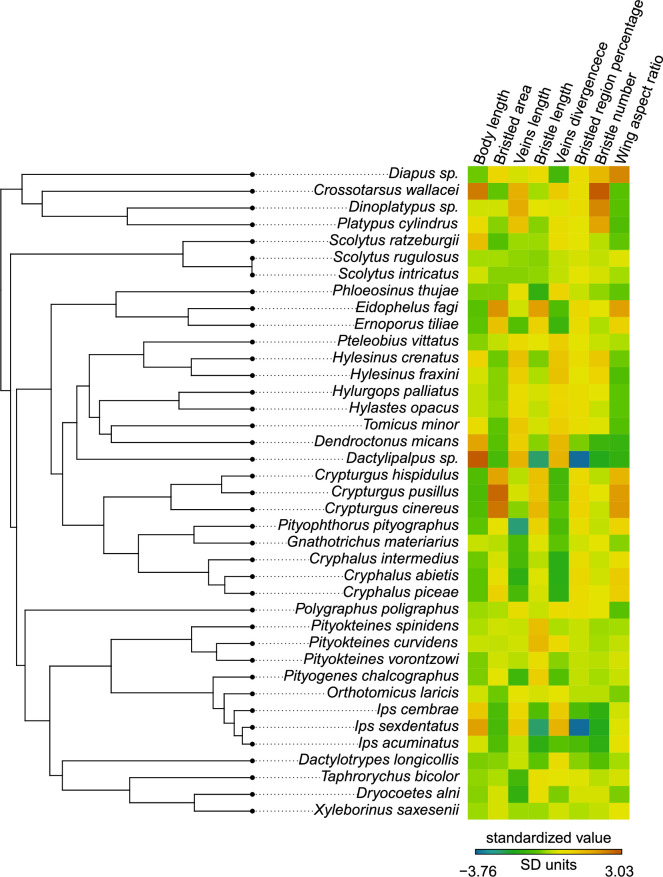
The phylogeny used in our phylogenetic comparative analyses. The heatmap shows species mean values for all wing traits (response variables) and body size (predictor variable) used in our SLOUCH analyses. The average trait values for species were standardized to the same scale (distance to the cross-species mean in standard deviations) to enable mapping on a single colour scale.

The number of bristles did not increase with decreasing body size, and the SLOUCH model for this trait was statistically insignificant (table 1). This feature displayed a very high degree of phylogenetic effect, suggesting that its adaptation potential is strongly influenced by ancestral stages (table 1). Notably, this pattern was evident during measurement, where closely related taxa showed clear similarity in their number of bristles. For instance, members of the subfamily Platypodinae exhibited consistently large numbers of marginal bristles despite variations in body size, while members of the genus *Ips* displayed significantly fewer bristles across different body sizes (figure 3).

The bristle length increased significantly as body size decreased (table 1, figure 2). This trait was strongly influenced by miniaturization and demonstrated a rapid adaptation rate, enabled by low evolutionary constraints (table 1; see the regression slope in figure 2). Bristle elongation appears most pronounced in the proximal wing region (figure 2), where the solid wing membrane was significantly reduced.

### (b) Wing shape and wing venation

The divergence of veins in the proximal wing region (within the medial field) decreased with body size reduction (table 1, figure 2). This indicates that the cubital and medial posterior wing veins move closer together during body miniaturization. The influence of miniaturization on this trait was strong (see the regression slope, figure 2), and the percentage of explained variance was considerable (*R*² = 0.50). Nevertheless, the trait experiences a significant level of phylogenetic constraint, requiring more than 19% of the group’s phylogenetic history to adapt halfway to its optimal stage (table 1, figure 2).

Relative vein length decreased significantly with progressive body miniaturization (table 1, figure 2). The trait has, however, evolved slowly owing to high evolutionary constraints (table 1; see the regression slope in (figure 2)). More than 80% of the group’s entire phylogenetic history would be required for this trait to adapt halfway to its optimal stage (table 1).

The wing aspect ratio increased significantly with body miniaturization (table 1, figure 2). Smaller species exhibited notably narrower proximal parts of the wings compared with larger taxa. The effect of miniaturization on this wing parameter was strong (see the regression slope, figure 2) and the percentage of explained variation was considerable (*R*² = 0.38, table 1). The evolutionary inertia associated with this trait was low, indicating that wing proportions (length to width) can adapt rapidly to new body-size regimes (table 1, figure 2).

## 4. Discussion

**Figure 4.**
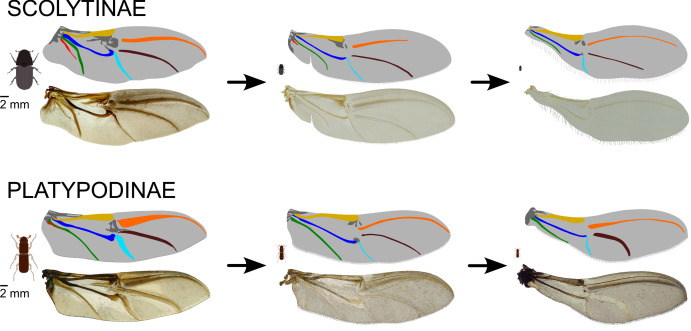
General patterns of wing modifications associated with body-size reduction in Scolytinae and Platypodinae (Coleoptera: Curculionidae).

For the first time, we have evaluated the relative roles of evolutionary constraints and functional demands in shaping the diversity of wing architecture in microinsects. Our phylogenetic comparative analysis of 39 beetle species with starkly different body sizes revealed that progressive miniaturization is accompanied by significant wing membrane reduction, marginal bristle development (length increase, coverage increase), wing narrowing and vein convergence and reduction (figure 4). Most importantly, we found that wing traits differ greatly in their capacity for rapid adaptation to body miniaturization (figure 2). Certain modifications, such as bristle elongation, wing narrowing and bristled region expansion, can evolve swiftly, requiring less than 8% of the clade’s phylogenetic history to optimize halfway to the functionally optimal stage (table 1, figure 2). In contrast, other wing modifications—including an increase in bristled area, vein length reduction and vein convergence—exhibit a much lower potential for rapid adaptation owing to strong evolutionary constraints, requiring over 18% of the group age (more than 18.9 Ma [34]) to adapt halfway to the optimal stage (table 1, figure 2). Evolutionary constraints were especially high in the case of vein length and bristled area, requiring 80.2% (84.2 Ma) and 107.4% (112.77 Ma), respectively, of the group’s phylogenetic history to adapt halfway to the optimal state (table 1, figure 2). These disparities in evolutionary rates across wing traits may help explain why unrelated microinsects exhibit striking convergence in certain wing features (e.g. elongated bristles, narrowed wings) while retaining significant divergence in others (e.g. wing venation development, proportion of bristled to membranous wing area).

### (a) Evolution of bristles

The progressive replacement of a solid wing membrane with an expanding bristled area is a key feature of ptiloptery [10,11]. Our findings indicate that this transformation primarily results from bristle elongation and the expansion of the bristled region. Short marginal bristles scattered over the bottom wing edge occur in most of the analysed bark beetle species, including much larger taxa. We did not detect a significant effect of body miniaturization on the number of bristles, which appears to be significantly influenced by ancestral stages (figure 3). For instance, members of the subfamily Platypodinae generally possess a large number of marginal bristles, whereas species within the genus *Ips* exhibit far fewer (figure 3). It should be noted that other studies indicated that bristle diameter and spacing play a more critical role in aerodynamic optimization than bristle number alone [10,37]. Wings with appropriately spaced bristles can function aerodynamically as solid surfaces, similar to conventional membranous wings of larger insects [37], which may explain why bristle number does not necessarily correlate with body size. Notably, some taxa (e.g. members of the subfamily Platypodinae) may even require a reduction in bristle number to achieve optimal spacing during body-size decrease. Alternatively, it can be speculated that marginal bristles might also play roles in other, not flight-related, functional contexts. However, further research is needed to identify the selective pressures shaping marginal bristle number in bark beetles.

Our analysis revealed a strong effect of miniaturization on the bristled area, underscoring the functional significance of this adaptation. The development of marginal bristles in microinsects provides several advantages. Bristled surfaces increase the effective wing surface area without adding substantial mass, which is crucial for small flyers [38] because it decreases the inertial cost of wing flapping [39]. Bristled wings may also help small insects cope with strong, shifting winds by minimizing sudden fluctuations in aerodynamic force compared with a solid surface [40]. Moreover, the bristled wing structure allows air to flow between the bristles during certain phases of the wing stroke while still generating only slightly less lift compared with solid wings. This combination of relatively high lift and significantly reduced drag results in a substantial improvement of the lift-to-drag ratio [4,10,41]. Finally, replacing solid membrane regions with bristles lowers wing mass, thereby enhancing the body-mass-specific aerodynamic power coefficient and reducing the resource demands of wing development [4,10]. Our results suggest that bristle elongation progresses more rapidly in wing regions where membrane reduction is most pronounced—particularly in the proximal region (e.g. anal field), suggesting an interesting adaptive compromise. Reducing the solid membrane area in the proximal part of the wing, where motion is slower and lift production is low, allows for a significant reduction in wing mass without a drastic loss of aerodynamic force. In contrast, modifications in the distal region of the wing appear more functionally limited. This is probably because the distal part moves faster and contributes largely to lift generation, and a solid wing surface is more effective than a bristled surface in generating aerodynamic force per unit area [4]. Nevertheless, we found that replacing a solid membrane with a bristled area requires considerable evolutionary time, which may explain why some groups of similar size but differing clade age show notable differences in this trait [11,14,41,42]. The shift from membranous to bristled wings seems to be a developmentally challenging transformation requiring coordinated changes across several modules of the wing (e.g. simultaneous membrane reduction, venation re-patterning and bristle architecture changes).

### (b) Wing shape and venation modifications

We found that miniaturization has a profound impact on wing shape and venation. As body size decreases, the proximal part of the wing becomes narrower, giving the wings of the smallest beetles a characteristic ‘racket-shaped’ appearance. Similar patterns occur in many microinsects, including wasps, other beetle groups and flies [11,16,43,44]. As in bark beetles, the distal part of the wing in these taxa becomes relatively wider than the narrowed proximal region, and the geometric centre of the wing shifts apically [11,16,43,44]. This modification is functionally crucial for microinsects because the further the wing area is distributed from the wing base, the greater the moment of area. During flapping flight, the distal regions of the wing move at higher speeds than the proximal regions, generating more aerodynamic force per unit area. Consequently, wings with a greater moment of area are associated with more efficient flapping flight compared with those with a lower moment of area [11,45]. Our findings indicate that these modifications in wing proportions can evolve rapidly owing to a low level of evolutionary constraints. This might explain the observed striking convergence in wing shape among distantly related microinsects, as such ‘racket-shaped’ adaptation can evolve relatively quickly, and it has great functional importance in all small flyers. Furthermore, we documented that the ‘racket-shaped’ wing appearance primarily results from a reduction in the bottom part of the proximal wing region, including the anal and medial fields. This reduction occurs through both the loss of wing membrane and the progressive convergence of veins, leading to a substantial decrease in both the anal and the medial wing fields. In the smallest analysed taxa of Platypodinae (mean body length = 1.88 mm), the wing blade is already highly modified, displaying a racket-shaped appearance. However, the marginal bristles are not significantly elongated compared with those in larger taxa (figure 4). This suggests that the selection pressure for wing blade narrowing occurs at earlier stages of wing miniaturization, while the pressure for bristle elongation emerges later.

We observed that the reduction and simplification of wing venation occur with progressive body miniaturization, a general pattern found in insects [3,10,11,13,18,46]. However, it is important to note that microinsects still exhibit significant among-group variation in wing venation, ranging from a complete absence of veins to the presence of several relatively well-preserved wing veins [11,47]. A synthesis of wing venation patterns across different insect orders [47] found no direct correlation between venation pattern and wing size, indicating that wings of vastly different sizes can share similar venation patterns. While some wing features may change rapidly with size, the fundamental elements of venation appear to be strongly conserved [16,48]. Our findings support this conclusion; although miniaturization leads to vein reduction, this process is largely constrained by strong evolutionary inertia. Vein convergence (probably finally leading to fusion) evolves much faster than vein shortening. The genetic regulatory networks responsible for insect vein development are highly conserved [47]. This suggests that even when selection favours vein length reduction, the entrenched genetic programme may significantly bias the outcome.

It should also be emphasized that insect wing morphology might be potentially shaped by other selection drivers that are not included in our analysis. It is widely known that wing morphology in many insect groups, including wasps and flies, can be strongly affected by specific adaptations to behaviours such as aerial courtship or guarding flight [1,10,16,18,48]. However, such behaviours are unknown and are rather unlikely to occur in bark beetles [19]. It can be speculated that bark beetle wings may be adapted for passive flight (e.g. wind dispersal), as passive long-distance dispersal is documented in certain species [19]. Nevertheless, even these taxa rely primarily on active flight to locate suitable host trees and mates.

## 5. Conclusion

Our study documents the transition from macro membranous wings to highly derived micro wings with significant signs of ptiloptery, revealing a complex interplay between functional demands and evolutionary constraints. While some adaptations—such as bristle elongation, wing narrowing or expansion of the bristled region—were found to evolve rapidly during body miniaturization, the modification of other traits, including venation development, vein divergence and the proportion of the bristled area, appears to be strongly constrained by developmental inertia. This disparity in the evolutionary rates of certain wing features constitutes a significant puzzle piece for understanding why miniaturized insects often converge on certain wing modifications while retaining substantial divergence in others. Our conclusions are drawn from two weevil clades (Scolytinae and Platypodinae) and should be tested across other insect lineages to assess their generality.

Despite the strong selective pressures imposed by significant body-size reduction, we found that certain structural elements of wing architecture persisted, indicating that deep-seated developmental constraints might moderate even powerful adaptive pressure to flight in a fundamentally different physical environment. The retention of ancestral traits (e.g. venation patterns), even in highly miniaturized species, underscores that evolutionary change is not simply a matter of selection optimizing biological structures to particular functions but rather the outcome of a dynamic balance between adaptation and constraints.

## Data Availability

All data and R code used in the analyses are publicly available on Dryad [49].
